# *BRCA1* mutation carriers have a lower number of mature oocytes after ovarian stimulation for IVF/PGD

**DOI:** 10.1007/s10815-017-1014-3

**Published:** 2017-08-22

**Authors:** I. A. P. Derks-Smeets, T. C. van Tilborg, A. van Montfoort, L. Smits, H. L. Torrance, M. Meijer-Hoogeveen, F. Broekmans, J. C. F. M. Dreesen, A. D. C. Paulussen, V. C. G. Tjan-Heijnen, I. Homminga, M. M. J. van den Berg, M. G. E. M. Ausems, M. de Rycke, C. E. M. de Die-Smulders, W. Verpoest, R. van Golde

**Affiliations:** 1 Department of Clinical Genetics, Maastricht University Medical Center, P.O. Box 5800, 6202 AZ Maastricht, The Netherlands; 2 GROW – School for Oncology and Developmental Biology, Maastricht University, P.O. Box 616, 6200 MD Maastricht, The Netherlands; 3 Department of Reproductive Medicine, University Medical Center Utrecht, P.O. Box 85500, 3508 GA Utrecht, The Netherlands; 4 Department of Obstetrics and Gynecology, Maastricht University Medical Center, P.O. Box 5800, 6202 AZ Maastricht, The Netherlands; 5 Department of Epidemiology, Maastricht University, P.O. Box 616, 6200 MD Maastricht, The Netherlands; 6 Department of Internal Medicine, Division of Medical Oncology, Maastricht University Medical Center, P.O. Box 5800, 6202 AZ Maastricht, The Netherlands; 7 Department of Obstetrics and Gynecology, University Medical Center Groningen, University of Groningen, P.O. Box 30.001, 9700 RB Groningen, The Netherlands; 8 Center for Reproductive Medicine, Academic Medical Center, P.O. Box 22660, 1100 DD Amsterdam, The Netherlands; 9 Department of Genetics, University Medical Center Utrecht, P.O. Box 85500, 3508 GA Utrecht, The Netherlands; 10 Center for Medical Genetics, Universitair Ziekenhuis Brussel, Laarbeeklaan 101, 1090 Brussels, Belgium; 11 Center for Reproductive Medicine, Universitair Ziekenhuis Brussel, Laarbeeklaan 101, 1090 Brussels, Belgium

**Keywords:** *BRCA1/2* mutations, Ovarian reserve, Mature oocytes, IVF, Preimplantation genetic diagnosis

## Abstract

**Purpose:**

The aim of this study was to determine whether *BRCA1/2* mutation carriers produce fewer mature oocytes after ovarian stimulation for in vitro fertilization (IVF) with preimplantation genetic diagnosis (PGD), in comparison to a PGD control group.

**Methods:**

A retrospective, international, multicenter cohort study was performed on data of first PGD cycles performed between January 2006 and September 2015. Data were extracted from medical files. The study was performed in one PGD center and three affiliated IVF centers in the Netherlands and one PGD center in Belgium. Exposed couples underwent PGD because of a pathogenic *BRCA1/2* mutation, controls for other monogenic conditions. Only couples treated in a long gonadotropin-releasing hormone (GnRH) agonist-suppressive protocol, stimulated with at least 150 IU follicle stimulating hormone (FSH), were included. Women suspected to have a diminished ovarian reserve status due to chemotherapy, auto-immune disorders, or genetic conditions (other than *BRCA1/2* mutations) were excluded. A total of 106 *BRCA1/2* mutation carriers underwent PGD in this period, of which 43 (20 *BRCA1* and 23 *BRCA2* mutation carriers) met the inclusion criteria. They were compared to 174 controls selected by frequency matching.

**Results:**

Thirty-eight *BRCA1/2* mutation carriers (18 *BRCA1* and 20 *BRCA2* mutation carriers) and 154 controls proceeded to oocyte pickup. The median number of mature oocytes was 7.0 (interquartile range (IQR) 4.0–9.0) in the BRCA group as a whole, 6.5 (IQR 4.0–8.0) in *BRCA1* mutation carriers, 7.5 (IQR 5.5–9.0) in *BRCA2* mutation carriers, and 8.0 (IQR 6.0–11.0) in controls. Multiple linear regression analysis with the number of mature oocytes as a dependent variable and adjustment for treatment center, female age, female body mass index (BMI), type of gonadotropin used, and the total dose of gonadotropins administered revealed a significantly lower yield of mature oocytes in the BRCA group as compared to controls (*p* = 0.04). This finding could be fully accounted for by the BRCA1 subgroup (*BRCA1* mutation carriers versus controls *p* = 0.02, *BRCA2* mutation carriers versus controls *p* = 0.50).

**Conclusions:**

Ovarian response to stimulation, expressed as the number of mature oocytes, was reduced in *BRCA1* but not in *BRCA2* mutation carriers. Although oocyte yield was in correspondence to a normal response in all subgroups, this finding points to a possible negative influence of the *BRCA1* gene on ovarian reserve.

**Electronic supplementary material:**

The online version of this article (doi:10.1007/s10815-017-1014-3) contains supplementary material, which is available to authorized users.

## Introduction

Contradicting results have been published on a potential influence of mutations in the *BRCA1* and *BRCA2* genes on ovarian reserve. Mutations in the *BRCA* genes are primarily known for their predisposition to breast and ovarian cancer [1]. The *BRCA* genes act as tumor suppressor genes and are involved in DNA double-strand break repair [2]. An impaired function leads to an accumulation of intracellular DNA damage. This may affect cellular growth mechanisms, leading to carcinogenic transformation [3]. Alternatively, accumulating DNA damage may induce growth arrest, leading to apoptosis [4]. Hypothetically, this may be illustrated in non-dividing cell populations, e.g., the ovarian follicle pool.

Oktay et al. [5] were the first to observe a reduced ovarian response to ovarian stimulation for in vitro fertilization (IVF) in *BRCA1* mutation-positive cancer patients undergoing fertility preservation. This was not confirmed by another report on the ovarian response to IVF stimulation in a combined group of *BRCA1/2* mutation carriers undergoing fertility preservation because of breast cancer and asymptomatic *BRCA1/2* mutation carriers undergoing IVF with preimplantation genetic diagnosis (PGD) [6]. Contradicting results have also been published when assessing ovarian reserve in *BRCA1/2* mutation carriers using other endpoints. Several studies on age of natural menopause reported an earlier menopause in both *BRCA1* and *BRCA2* mutation carriers [7–9]. The majority of studies using anti-Müllerian hormone (AMH) as an indicator for the number of (pre-)antral follicles in the ovaries detected lower levels of AMH in *BRCA1* mutation carriers, not in *BRCA2* mutation carriers [10–13]. Studies using several other reproductive outcome parameters (e.g., parity) did not point to a reduced fecundity in *BRCA1/2* mutation carriers [14–18].

Ovarian response to stimulation for IVF is a strong indicator for ovarian reserve status [19]. Sufficient ovarian response is particularly important in PGD, where transfer criteria primarily involve genetic results. After a second selection on embryo quality, only a minority of the obtained embryos will be available for transfer. If a mutation in the *BRCA1* and/or *BRCA2* gene is associated with a lower ovarian reserve, this may have a negative effect on success chances of mutation carriers undergoing IVF for infertility reasons, for fertility preservation, as well as for PGD. PGD for *BRCA1/2* mutations has been performed for a decade now and the number of couples treated each year has been growing steadily [20, 21].

The objective of the current study is to clarify whether *BRCA1/2* mutation carriers produce less mature oocytes after ovarian stimulation for IVF/PGD.

## Material and methods

A retrospective, observational cohort study was carried out in five centers: Maastricht University Medical Center (center 1) and affiliated IVF centers University Medical Center Utrecht (center 2), University Medical Center Groningen (center 3), and Academic Medical Center Amsterdam (center 4), united in the Dutch consortium for PGD, and Universitair Ziekenhuis Brussel, Brussels, Belgium (center 5). The study period lasted from the introduction of PGD for hereditary cancer syndromes (i.e., 2006 for Brussels and 2008 for The Netherlands) until September 2015.

The exposed group consisted of couples who underwent IVF/PGD because of a pathogenic mutation in the *BRCA1* or *BRCA2* gene in the female (the “BRCA group”). All mutations were proven pathogenic by means that they had a verified significant disturbing effect on protein translation. The control group consisted of couples who underwent PGD because of an autosomal dominant or recessive disorder not known to be associated with a reduced ovarian reserve. For the selection of controls, frequency matching was used: control couples were selected blinded for outcome, based on treatment center and treatment period in order to obtain an equal distribution in both groups [22]. For this purpose, a chronological overview of PGD treatments performed per PGD center for autosomal dominant and recessive disorders (excluding conditions known for a (potential) effect on ovarian reserve (e.g., fragile X syndrome, myotonic dystrophy type 1) and male *BRCA1/2* mutation carriership) was created. Matching was done per PGD center: PGD treatments for female *BRCA1/2* mutations were identified, and (if available) four PGD treatments for autosomal dominant or recessive disorders chronologically performed closely before or after the PGD treatment for *BRCA1/2* were included as controls. In order to rule out bias from repetitive cycles, only first treatment cycles were included. First cycles with and without oocyte pick-up were included in order to assess the cancelation rate because of poor ovarian response in both groups.

Only treatments in a long gonadotropin-releasing hormone (GnRH) agonist-suppressive protocol, with stimulation with at least 150 IU follicle stimulating hormone (FSH) or human menopausal gonadotropin (hMG) per day, were included in order to obtain a homogenous study population with optimal ovarian stimulation [23]. Other inclusion criteria for both groups were: female age < 43 years, female body mass index (BMI) < 35 kg/m^2^, and female endogenous FSH < 15 IU/l. Exclusion criteria were a history of invasive (breast) cancer up to 2 years prior to IVF/PGD treatment, ovarian surgery, chemotherapy, pelvic radiation, polycystic ovary syndrome that conforms the Rotterdam criteria [24], and known endocrine, autoimmune, or genetic abnormalities (potentially) associated with a reduced ovarian reserve (e.g., fragile X premutation carriers, myotonic dystrophy type 1).

Final oocyte maturation was induced when sufficient dominant follicles were seen at ultrasound (i.e., at least four follicles > 14 mm in the Netherlands and at least three follicles > 17 mm in Brussels). The number of mature oocytes was assessed at the moment of intracytoplasmic sperm injection (ICSI). ICSI was used for fertilization in order to avoid contamination of the zona pellicuda with residual spermatozoa. Embryo biopsy was performed on day 3 after fertilization. Single-cell analysis of the removed blastomeres was performed using multiplex polymerase chain reaction (PCR), as described elsewhere [20, 25, 26]. Data were extracted from medical files.

### Ethical approval

The study was approved by the Institutional Review Boards of Maastricht University Medical Center (METC 14-4-163) and Universitair Ziekenhuis Brussel (2014/383). All couples gave their written informed consent for IVF/PGD treatment, and the usage of their PGD data for scientific research before the treatment was started.

### Statistical analysis

Patient characteristics and outcome data are presented as mean and standard deviation, median and interquartile range (IQR), or frequency and percentage, depending on the distribution of the variable. Where outcome data were not normally distributed, bivariate analyses were performed using non-parametric tests (Mann-Whitney *U* test). A linear regression model was used to assess an association between *BRCA1/2* mutation status and ovarian response in terms of the number of obtained mature oocytes. The number of mature oocytes was transformed using the square root, in order to obtain an approximately normal distribution of the residuals. Adjustments were made for potential confounding factors, i.e., treatment center, female age, female BMI, type of gonadotropin administered (FSH or hMG), and total dose of gonadotropin administered. These factors were incorporated because of a potential negative influence of an advanced age, higher BMI, and the use of hMG on the number of mature oocytes yielded and because an effect of the treatment center and the cumulative dose of gonadotropins applied could not be ruled out. Age and BMI were both assessed as continuous and categorical variables (age ≤ 30 versus > 30 years, age ≤ 35 versus > 35 years, BMI ≤ 25 versus BMI > 25). Subgroup analyses were conducted to determine potential differences in the primary outcome between *BRCA1* mutation carriers and the control group and *BRCA2* mutation carriers and the control group. A sensitivity analysis was performed excluding center 5, since this center used the long agonist protocol particularly for expected poor responders. Statistical analyses were performed using SAS statistical analysis software for Windows, version 9.3.

The study was powered on a previously reported difference in obtained oocytes following IVF in *BRCA* carriers (7.9 (95% CI 4.6–13.8) oocytes in *BRCA* mutation carriers compared to 11.3 (95% CI 9.1–14.1) oocytes in women without a *BRCA* mutation [5]). The inclusion of 50 *BRCA* mutation carriers and 200 controls would be sufficient to detect a difference of the aforementioned magnitude, with alpha set at 0.05 and beta at 0.8.

## Results

### Patient characteristics

In total, 106 female *BRCA1/2* mutation carriers underwent PGD in the study period, of whom 66 (62.3%) had a *BRCA1* mutation and 40 (37.7%) a *BRCA2* mutation. Twelve carriers had a history of invasive breast cancer and chemotherapy (nine *BRCA1* and three *BRCA2* mutation carriers), and 51 carriers were excluded for other reasons (Table 1). Of the 43 included carriers, 20 (46.5%) had a *BRCA1* mutation and 23 (53.5%) a *BRCA2* mutation. Of the 174 controls, 119 (68.4%) underwent PGD because of an autosomal dominant condition and 55 (31.6%) because of an autosomal recessive condition (Table 2). An overview of the distribution of the couples over the five centers is provided in supplemental Table 1.

**Table 1:** The number of eligible women and the reasons for exclusion

	BRCA group, *n* = 106	Control group, *n* = 174
Reason for exclusion (*n*)		
Breast cancer + chemotherapy	12^a^	
Endocrine/autoimmune disorder	5^b^	
Polycystic ovarian syndrome	3	
Ovarian surgery	1	
Other genetic conditions	1^c^	
Regular IVF prior to PGD	0	
Different IVF protocols^d^	36	
Only cycles with <150 IU FSH per day	5	
First cycles included (*n*)	43	174
Cancel in the first cycle (*n*, %)	5/43 (11.6)	20/174 (11.5)
First cycles with oocyte pickup (*n*, %)	38/43 (88.4)	154/174 (88.5)

*IVF* in vitro fertilization, *PGD* preimplantation genetic diagnosis, *FSH* follicle stimulating hormone

^a^Five of these women were also treated in an IVF protocol other than a long GnRH agonist-suppressive protocol

^b^Two of these women were also treated in an IVF protocol other than a long GnRH agonist-suppressive protocol

^c^Female *CHEK2* mutation

^d^Treatment in an IVF protocol other than a long GnRH agonist-suppressive protocol

**Table 2:** Patient characteristics

	BRCA group, *n* = 43	Control group, *n* = 174
Female age (mean, SD)	31.4 ± 3.7	32.1 ± 4.1
Female BMI (mean, SD)	23.8 ± 3.0	23.9 ± 3.5
AD disorders (*n*, %)	43 (100.0)	119 (68.4)
Female carriers	42	59^a^
Male carriers	n/a	57
Both partners	1	3
*BRCA1* (*n*, %)	20 (46.5)	n/a
*BRCA2* (*n*, %)	22 (51.2)	n/a
*BRCA2* female + retinoblastoma male (*n*, %)	1 (2.3)	n/a
Huntington’s disease	n/a	25^b^
Neurofibromatosis type 1	n/a	12^c^
Myotonic dystrophy type 1^d^	n/a	10
Familial adenomatous polyposis	n/a	10
Spinocerebellar ataxia type 3	n/a	8
Marfan syndrome	n/a	7
Other	n/a	47^e^
AR disorders (*n*, %)	n/a	55 (31.6)
Cystic fibrosis	n/a	16
Spinal muscular atrophy	n/a	13^e^
Other	n/a	26

*SD* standard deviation, *BMI* body mass index, *AD* autosomal dominant, *AR* autosomal recessive, *n/a* not applicable

^a^One woman had both Peutz-Jeghers syndrome and porencephalia

^b^Of which five couples opted for exclusion PGD

^c^Of which two couples had two indications for PGD

^d^Only males with myotonic dystrophy type 1 were included, since myotonic dystrophy type 1 is potentially associated with a reduced ovarian reserve

^e^Of which one couple had two indications for PGD

### Bivariate analyses

Thirty-eight out of 43 BRCA cycles and 154 out of 174 control cycles proceeded to oocyte pick-up. The cancelation rate due to a poor response was 3/43 (7.0%) in the BRCA group and 16/174 (9.3%) in the control group (*p* = 0.35). The median number of cumulus oocyte complexes was 9.0 (IQR 5.8–11.0) and 10.0 (IQR 7.0–14.0) in the BRCA and control group, respectively (*p* = 0.05, Table 3). The median number of mature oocytes was 7.0 (IQR 4.0–9.0) and 8.0 (IQR 6.0–11.0, *p* = 0.02), respectively. The observed difference in mature oocytes could be fully accounted to women with a *BRCA1* mutation: *BRCA1* mutation carriers (*n* = 18) produced a median of 6.5 (IQR 4.0–8.0) mature oocytes, compared to 8.0 (IQR 6.0–11.0) in the control group (*p* = 0.01). This difference was not observed in the BRCA2 subgroup (*n* = 20, median 7.5 (IQR 5.5–9.0) in the BRCA2 subgroup, *p* = 0.20).

**Table 3:** First IVF/PGD cycles

	BRCA group (*n* = 38)	Control group (*n* = 154)	*p* value	BRCA1 subgroup (*n* = 18)	*p* value^a^	BRCA2 subgroup (*n* = 20)	*p* value^a^
Cumulative dose of exogenous FSH administered (median, IQR)^b^	1987.5 (1762.5–2812.5)	1950.0 (1650.0–2575.0)	0.65	1950.0 (1650.0–2550.0)	0.98	2137.5 (1800.0–3356.3)	0.49
Cumulus oocyte complexes (median, IQR)^b^	9.0 (5.8–11.0)	10.0 (7.0–14.0)	0.05	8.5 (5.0–11.3)	0.13	9.0 (6.0–10.8)	0.14
Mature oocytes (median, IQR)^b^	7.0 (4.0–9.0)	8.0 (6.0–11.0)	0.02	6.5 (4.0–8.0)	0.02	7.5 (5.5–9.0)	0.20
FSH/mature oocyte (median, IQR)^b^	353.0 (210.7–521.9)	250.0 (168.6–375.0)	0.03	383.0 (208.3–521.9)	0.06	326.3 (203.6–600.0)	0.14
Fraction of normally fertilized oocytes (2PN) per injected oocyte (median, IQR)^b^	0.7 (0.7–0.8)	0.7 (0.6–0.9)	0.89	0.8 (0.7–0.8)	0.46	0.7 (0.6–0.8)	0.62
Fraction of embryos biopsied for PGD per injected oocyte (median, IQR)^b^	0.7 (0.6–0.8)	0.7 (0.6–0.8)	0.63	0.8 (0.7–0.8)	0.21	0.7 (0.5–0.8)	0.63
Fraction of aneuploid embryos per injected oocyte (median, IQR)^b,c^	0.0 (0.0–0.1)	0.1 (0.0–0.2)	0.04	0.1 (0.0–0.2)	0.95	0.0 (0.0–0.0)	0.00
Cycles with embryonic transfer (*n*, %)^d^	34/38 (89.5)	129/154 (83.8)	0.38	17/18 (94.4)	0.23	17/20 (85.0)	0.89
Pregnancy with fetal heart beat at 7 weeks of gestation (*n*, %)^d,e^	10/34 (29.4)	39/129 (30.2)	0.93	3/17 (17.6)	0.28	7/17 (41.2)	0.36

*IVF* in vitro fertilization, *PGD* preimplantation genetic diagnosis, *FSH* follicle stimulating hormone, *IQR* interquartile range, *PN* pronuclei

^a^Compared to the control group

^b^Analyzed using the Mann-Whitney *U* test

^c^Aneuploid for the chromosome analyzed during PGD

^d^Analyzed using the chi-square test

^e^Only cycles included which resulted in embryonic transfer

There was no difference in the cumulative dose of exogenous FSH administered between groups (1987.5 IU (IQR 1762.5–2812.5 IU) in the BRCA group as a whole, 1950.0 IU (IQR 1650.0–2550.0 IU) in the BRCA1 subgroup, 2137.5 IU (IQR 1800.0–3356.3 IU) in the BRCA2 subgroup, and 1950.0 IU (IQR 1650.0–2575.0 IU)) in controls (all *p* > 0.05, Table 3). As the number of mature oocytes was lower in the BRCA group, we explored whether the ratio of administered FSH per obtained mature oocyte obtained was higher in this group (i.e., whether *BRCA* mutation carriers needed more FSH to obtain the same amount of oocytes and/or produced less oocytes when the same dose of FSH was applied). In the BRCA group as a whole, more FSH was administered per obtained mature oocyte when compared to the control group (median FSH/mature oocyte ratios 353.0 (IQR 210.7–521.9) and 250.0 (IQR 168.6–375.0), respectively, *p* = 0.03). The FSH/mature oocyte ratio was highest in the BRCA1 subgroup (median FSH/mature oocyte ratio 383.0 (IQR 208.3–521.9) in the BRCA1 subgroup and 326.3 (IQR 203.6–600.0) in the BRCA2 subgroup). The fraction of normally fertilized oocytes (2PN oocytes) was comparable between groups (Table 3). The pregnancy rate was lower in women with a *BRCA1* mutation, but this did not reach significance.

### Multivariable analyses

Linear regression analyses with the square root transformed number of mature oocytes as the dependent variable showed that the difference in the number of mature oocytes between the BRCA group and control group remained statistically significant after adjustment for treatment center, female age, female BMI, type of gonadotropin (FSH or hMG), and cumulative dose of FSH administered (*p* = 0.04, Table 4). Again, this difference was only present in *BRCA1* mutation carriers as compared to controls (*p* = 0.02), not in *BRCA2* mutation carriers (*p* = 0.50).

**Table 4:** Multivariable analyses

	Number of mature oocytes (linear regression analysis)		
	Β	SE	*p*
*BRCA1/2* vs. controls	−0.28	0.13	0.04
*BRCA1* vs. controls	−0.45	0.18	0.02
*BRCA2* vs. controls	−0.12	0.17	0.50

Adjusted for treatment center, female age, female body mass index, type of gonadotropin used, and total dosage of gonadotropins administered

Additional analyses were performed to allow for a possible non-linear effect of female age and female BMI on the number of mature oocytes, by introducing these variables as a dichotomous (instead of linear) variable in the multivariable model (age ≤ 30 versus > 30 years, age ≤ 35 versus > 35 years, and BMI ≤ 25 versus BMI > 25). This did not change the outcome. A sensitivity analysis excluding center 5 (as stated above, the fact that in this center the long agonist protocol was primarily used for expected poor responders could have introduced bias) did neither change the outcome.

## Discussion

In this study, a lower number of mature oocytes was found in women with a *BRCA1* mutation in response to ovarian stimulation for IVF/PGD.

Diverse studies have been reported on a possible diminished ovarian reserve in *BRCA* mutation carriers, using different primary outcomes and study designs. Oktay et al. [5] were the first to report a lower yield of oocytes in eight *BRCA1*, but not in four *BRCA2*-mutated breast cancer patients. A case-control study by Shapira et al. [6] found no difference in oocyte yield according to *BRCA* mutation status in 62 *BRCA* mutation-positive women. However, the inclusion of cancer patients and patients stimulated in different IVF protocols and the lack of clarity regarding minimal stimulation doses applied may have obscured an existing difference.

Previous studies on ovarian reserve in *BRCA1/2* mutation carriers using non-IVF-related parameters did not show consistent results. It is challenging, however, to study ovarian reserve in *BRCA1/2* mutation carriers because of the presence of several confounding factors in this particular population. Firstly, breast cancer [27, 28] as well as its potential gonadotoxic treatment [29] has a negative effect on ovarian reserve. Secondly, many *BRCA1/2* mutation carriers opt for a risk-reducing salpingo-oophorectomy. The timing of this event may be influenced by personal cancer history and related to the menopausal transition. As a consequence, studies on age at natural menopause in *BRCA1/2* mutation carriers have important limitations, as set out by van Tilborg et al. [30]. Two studies reported a younger age of natural menopause in both *BRCA1* and *BRCA2* mutation carriers [8, 9]. A third study found a younger age of menopause in *BRCA1* mutation carriers with and without breast cancer [7]. Two other studies did not detect a difference in the age of natural menopause between carriers and non-carriers [30, 31]. Studies were troubled by both the inclusion [7] and exclusion of breast cancer patients [8], the exclusion of women who experienced menopause due to other reasons than natural menopause [8], bias resulting from informative censoring due to risk-reducing salpingo-oophorectomy uptake [9], the inclusion of only few women who had actually reach natural menopause [31], and/or other forms of bias [30]. Three studies have found a lower AMH in *BRCA1* mutation carriers and not in *BRCA2* mutation carriers [10, 12, 13], while two other studies did not detect a difference between *BRCA1* and *BRCA2* mutation carriers and controls [11, 32]. Differences in outcome may be the result of variances in study design, in particular the inclusion of breast cancer patients [10] and women with irregular menstrual cycles and/or polycystic ovarian syndrome [11–13], the lack of appropriate adjustment for potential confounding factors in the analysis [10, 11], and/or power issues [32]. Pregnancy rate and parity in *BRCA1/2* mutation carriers were not different from controls [14–16]. Some studies even report more pregnancies and children born per mother among *BRCA1/2* mutation carriers [17, 18].

Our study provides additional evidence for a reduced ovarian reserve in *BRCA1* mutation carriers, although the effect size was rather small and the oocyte yield was in the range of a normal response for all subgroups. Consequently, our finding may be more interesting from a biological point of view than relevant for clinical practice. The strengths of our study are (a) the large homogeneous cohort of *BRCA1/2* mutation carriers without recent malignant disease [27, 28], (b) the use of the same IVF protocol including only first cycles, and (c) the application of frequency matching, resulting in a representative control group. Our study also has limitations, mainly associated with the retrospective study design although the most important outcome data were complete for all inclusions (supplemental Table 2). Firstly, during the study period, different IVF protocols were used in the participating centers. In order to obtain a homogenous stimulated cohort, we only included couples treated in a long GnRH agonist-suppressive protocol with at least 150 IU gonadotropins per day. This selection led to a smaller cohort than initially powered. Nevertheless, the effect size in the *BRCA1* subgroup was large enough to be detectable. Additionally, this strategy may have introduced bias due to the exclusion of expected hyperresponders (treated with lower doses of FSH per day) and the inclusion of an excess of suspected poor responders, since in center 5, this IVF protocol was only the first choice in this subgroup of patients. However, this may have had an effect on both the BRCA and control groups and a sensitivity analysis excluding center 5 did not change the primary outcome. Secondly, since the poor response rate was (non-significantly) higher in the control group, this could have biased our primary outcome. Thirdly, we did not have the opportunity to correct for lifestyle factors (e.g., smoking). Finally, the BRCA1 and BRCA2 subgroups were still relatively small. Consequentially, the absence of an effect of *BRCA2* dysfunction on ovarian response may have been the result of insufficient power.

Despite these limitations, our finding of an impaired response to ovarian stimulation in *BRCA1* mutation carriers and not in *BRCA2* mutation carriers is interesting and confirms several previous studies. The absence of a(n) (detectable) effect of *BRCA2* dysfunction on ovarian reserve in most studies may be the result of a true lack of a difference, of insufficient power, and/or of either a later-in-life-occurring or more subtle decline in ovarian reserve, corresponding to the lower risk and higher age at diagnosis of breast and ovarian cancer associated with *BRCA2* mutations [33]. Both *BRCA* genes are involved in DNA double-strand break repair, but their biological functions differ. The association between *BRCA1* and *BRCA2* and (reproductive) aging is demonstrated by the involvement of the *BRCA* genes in telomere maintenance: telomeres shorten with age [34, 35]. In human oocytes, DNA double-strand breaks are more prevalent with increasing age, while *BRCA1* expression is reduced by then [10]. *BRCA1* plays an important role in meiotic spindle formation in mice, and *BRCA1* mutant mice had fewer primordial follicles, produced fewer oocytes in response to ovarian stimulation, had a smaller litter size, and showed more DNA double-strand breaks in their oocytes with increasing age than wild-type mice [10, 36]. *BRCA2* dysfunction in mice has been associated with insufficient spermatogenesis, a depletion of germ cells in female mice, and a higher frequency of nuclear aberrations in mutant oocytes [37]. However, the involvement of *BRCA2* in DNA double-strand break repair is probably less comprehensive than *BRCA1* involvement [38]. Consequentially, it can be hypothesized that the effect of *BRCA2* dysfunction on ovarian reserve is less powerful than the effect of a *BRCA1* mutation and potentially only becomes visible at increasing age.

If *BRCA1/2* mutation carriers are affected with a reduced ovarian reserve, this might have several clinical consequences, such as a higher need for fertility treatment, a worse treatment outcome, an urge for more treatment attempts, and/or higher doses of fertility drugs. However, the size of the effect found in our study is probably too small to be of clinical relevance. Future clinical and molecular studies are needed to provide more insight into the role of the *BRCA(1)* gene(s) in the maintenance of the ovarian pool.

## Conclusions

A reduced yield of mature oocytes was found in *BRCA1* mutation carriers undergoing IVF/PGD, suggesting a role of the *BRCA1* gene in the maintenance of ovarian reserve.

## Electronic supplementary material

Table S1 (DOCX 22 kb).

Table S2 (DOCX 20 kb).
