# Supplementary material for: BRCA1 mutation carriers have a lower number of mature oocytes after ovarian stimulation for IVF/PGD
Source: J Assist Reprod Genet. 2017 Aug 22;34(11):1475–82. doi: 10.1007/s10815-017-1014-3 (PMC5699993; doi:10.1007/s10815-017-1014-3)
Supplement: Supplementary file 1 — (DOCX 22 kb). [file 10815_2017_1014_MOESM1_ESM.docx]

**Supplemental table 1: Inclusions per PGD center**

|  | Center 1 | Center 2 | Center 3 | Center 4 | Center 5 |
| --- | --- | --- | --- | --- | --- |
| **Inclusions**    **BRCA**  **Control** | **72**    **15**  **57** | **60**    **13**  **47** | **30**  **6**  **24** | **14**    **3**  **11** | **41**    **6**  **35** |
| Age (mean, SD) | 31.6 ± 3.8 | 32.1 ± 3.5 | 30.7 ± 4.0 | 30.7 ± 5.5 | 33.7 ± 4.1 |
| BMI (mean, SD) | 24.5 ± 3.0 | 24.2 ± 3.6 | 23.7 ± 3.3 | 23.7 ± 3.9 | 22.6 ± 3.2 |
| Couples with cancel in 1^st^ cycle due to poor response (n, %)  BRCA (n, %)    Control (n, %) | 10/72 (13.9%)  1/15 (6.7%)  9/57 (15.8%) | 6/60 (10.0%)  1/13 (7.7%)  5/47 (10.6%) | 2/30 (6.7%)  1/6 (16.7%)  1/24 (4.2%) | 1/14 (7.1%)  0/3 (0.0%)  1/11 (9.1%) | 0/41 (0.0%)  0/6 (0.0%)  0/35 (0.0%) |
| **First cycles with oocyte pick-up**  **BRCA**  **Control** | **59**  **13** (22.0%)  **46** (78.0%) | **53**  **11** (20.8%)  **42** (79.2%) | **28**  **5** (17.9%)  **23** (82.1%) | **13**    **3** (23.1%)  **10** (76.9%) | **39**  **6** (15.4%)  **33** (84.6%) |
| Type of gonadotropin  FSH  hMG  Missing | 44 (74.6%)  9 (15.2%)  6 (10.2%) | 52 (98.1%)  1 (1.9%)  0 (0.0%) | 16 (57.1%)  12 (42.9%)  0 (0.0%) | 0 (0.0%)  13 (100.0%)  0 (0.0%) | 5 (12.8%)  34 (87.2%)  0 (0.0%) |
| Cumulative FSH dose (IU)  (median, IQR) | 2250.0  (1950.0-3150.0) | 1600.0  (1500.0-1950.0) | 2250.0  (1950.0-2925.0) | 1800.0  (1650.0-2025.0) | 2250.0  (1800.0-2475.0) |
| Mature oocytes ~~1~~^~~st~~^ ~~cycle~~  (median, IQR) | 7.0  (5.0-9.0) | 8.0  (5.0-10.5) | 9.0  (6.3-11.5) | 8.0  (4.0-14.5) | 9.0  (6.0-11.0) |
| Pregnancy with fetal heart beat at 7 weeks ~~following 1~~^~~st~~^ ~~cycle~~    BRCA  Control | 14/59 (23.7%)  3/13 (23.1%)  11/46 (23.9%) | 13/53 (24.5%)  2/11 (18.2%)  11/42 (26.2%) | 3/28 (10.7%)  0/5 (0.0%)  3/23 (13.0%) | 4/13 (30.8%)  1/3 (33.3%)  3/10 (30.0%) | 15/39 (38.5%)  4/6 (66.7%)  11/33 (33.3%) |

PGD=preimplantation genetic diagnosis, SD=standard deviation, BMI=body mass index, FSH=follicle stimulating hormone, hMG=human menopausal gonadotropin, IU=international units, IQR=interquartile range
