# Supplementary material for: BRCA1 mutation carriers have a lower number of mature oocytes after ovarian stimulation for IVF/PGD
Source: J Assist Reprod Genet. 2017 Aug 22;34(11):1475–82. doi: 10.1007/s10815-017-1014-3 (PMC5699993; doi:10.1007/s10815-017-1014-3)
Supplement: Supplementary file 2 — (DOCX 20 kb). [file 10815_2017_1014_MOESM2_ESM.docx]

**Supplemental table 2: Missing data of included first cycles**

|  | BRCA1 subgroup | BRCA2 subgroup | Control group |
| --- | --- | --- | --- |
| BRCA mutation | 0 | 0 | n/a |
| Treatment center | 0 | 0 | 0 |
| Female age | 0 | 0 | 0 |
| Female BMI | 0 | 0 | 0 |
| Type of gonadotropin | 1 | 0 | 5 |
| Cumulative dose of exogenous FSH administered | 0 | 0 | 0 |
| Cumulus oocyte complexes | 0 | 0 | 0 |
| Mature oocytes | 0 | 0 | 0 |
| FSH/mature oocyte | 0 | 0 | 0 |
| Normally fertilized oocytes (2 PN) | 0 | 0 | 7 |
| Embryos biopsied for PGD | 0 | 0 | 1 |
| Aneuploid embryos | 0 | 0 | 2 |
| Lost to follow-up | 0 | 0 | 0 |

BMI=body mass index, FSH=follicle stimulating hormone, PN=pronuclei, PGD=preimplantation genetic diagnosis
